# Dynamics of humoral immune response in SARS-CoV-2 infected individuals with different clinical stages

**DOI:** 10.3389/fimmu.2022.1007068

**Published:** 2022-11-14

**Authors:** Yorjagis Mendez-Cortina, Ana Lucía Rodriguez-Perea, Mateo Chvatal-Medina, Tulio Jose Lopera, Natalia Alvarez-Mesa, Jan Karlo Rodas-Marín, Diana Carolina Moncada, Maria Teresa Rugeles, Paula Andrea Velilla

**Affiliations:** ^1^ Grupo Inmunovirología, Facultad de Medicina, Universidad de Antioquia, Medellín, Colombia; ^2^ Grupo de Investigación Hospital Alma Máter de Antioquia, Área de Investigación e Innovación, Hospital Alma Máter de Antioquia, Medellín, Colombia; ^3^ Infectious Diseases, Internal Medicine, Hospital Universitario San Vicente Fundación, Medellín, Colombia; ^4^ Facultad de Medicina, Universidad de Antioquia, Medellín, Colombia

**Keywords:** COVID-19, B cells, memory B cells, naïve B cells, neutralizing antibodies, disease severity

## Abstract

**Background:**

The COVID-19 pandemic remains a global health problem. As in other viral infections, the humoral immune response against SARS-CoV-2 is thought to be crucial for controlling the infection. However, the dynamic of B cells in the clinical spectrum of this disease is still controversial. This study aimed to characterize B cell subsets and neutralizing responses in COVID-19 patients according to disease severity through a one-month follow-up.

**Methods:**

A cohort of 71 individuals with SARS-CoV-2 infection confirmed by RT-PCR were recruited and classified into four groups: i) asymptomatic; ii) symptomatic outpatients; iii) hospitalized in ward, and iv) intensive care unit patients (ICU). Samples were taken at days 0 (inclusion to the study), 7 and 30. B cell subsets and neutralizing antibodies were assessed using multiparametric flow cytometry and plaque reduction neutralization, respectively.

**Results:**

Older age, male gender and body mass index over 25 were common factors among hospitalized and ICU patients, compared to those with milder clinical presentations. In addition, those requiring hospitalization had more comorbidities. A significant increase in the frequencies of CD19^+^ cells at day 0 was observed in hospitalized and ICU patients compared to asymptomatic and symptomatic groups. Likewise, the frequency of plasmablasts was significantly increased at the first sample in the ICU group compared to the asymptomatic group, but then waned over time. The frequency of naïve B cells decreased at days 7 and 30 compared to day 0 in hospitalized and ICU patients. The neutralizing antibody titers were higher as the severity of COVID-19 increased; in asymptomatic individuals, it was strongly correlated with the percentage of IgM^+^ switched memory B cells, and a moderate correlation was found with plasmablasts.

**Conclusion:**

The humoral immune response is variable among SARS-CoV-2 infected people depending on the severity and time of clinical evolution. In severe COVID-19 patients, a higher plasmablast frequency and neutralizing antibody response were observed, suggesting that, despite having a robust humoral immunity, this response could be late, having a low impact on disease outcome.

## Introduction

Coronavirus Disease 2019 (COVID-19) pandemic is caused by severe acute respiratory syndrome coronavirus-2 (SARS-CoV-2). To date, there have been 572 million reported cases worldwide, and nearly 6.5 million have succumbed to the disease (1). Therefore, it is imperative to understand the immune response underlying SARS-CoV-2 infection to generate therapeutic and preventive strategies. The clinical picture can range from asymptomatic to severe illness, where critical patients may course with acute respiratory distress syndrome (ARDS) requiring mechanical ventilation and intensive care unit (ICU) admission (2, 3). This heterogeneous response observed in COVID-19 participants has been attributed to both viral and host factors. Indeed, it is known that viral proteins such as ORF3b and NSP-3 blocked IFN-I pathway and NSP-1, -10, and -16 shutdown host mRNA translation machinery thus contributing to viral pathogenesis (4). Furthermore, viral evolution has been concentrated in Spike protein, with mutations conferring higher affinity to the cellular receptor, therefore the new variants seem to be more transmissible, ability to evade immunity but its less virulent (5, 6). Several host factors have been associated with higher severity as age, gender, presence of comorbidities, and magnitude and characteristics of the innate and adaptive immune responses (7).

The humoral immune response generated by the activation of B cells, production of neutralizing antibodies and generation of memory B cells is critical for the control of the infection, for preventing reinfections and for an effective response to vaccination. In the setting of SARS-CoV-2 infection, there is controversial evidence regarding the response of B cells and their subpopulations, as well as the production of neutralizing antibodies. It has been documented that the frequencies of CD19^+^ B cells are augmented in severe patients compared to mild patients and healthy donors (8). Inside the pool of circulating B lymphocytes, transitional B cells that are at immature stages before they migrate to the spleen, and naïve but mature B cells have been found to be decreased in severe, compared to mild COVID-19 individuals (9). However, researchers such as Rajamanickam et al. found an increase in the frequencies of this subset in severe cases compared to mild cases (10). As it has been demonstrated, after exposure to SARS-CoV-2, plasmablasts (PBs) are generated and are mainly augmented among critical individuals compared to uninfected ones, but decreased compared to mild COVID-19 individuals (8, 10, 11). Although memory B cell (MBC) subsets such as class-switched (IgD^-^CD27^+^) and not class-switched (IgD^+^CD27^+^) appear to be decreased in severe COVID-19 compared to mild individuals (8, 10), other authors have reported an increase in activated and resting MBCs (9). Moreover, SARS-CoV-2-specific MBCs augment initially and are maintained for up to 4-5 months after infection (12).

The antibody response is heterogeneous among COVID-19 individuals, and not all patients develop neutralizing antibodies (13, 14). Spike (S)-specific neutralizing antibodies have been positively correlated with the severity of infection when evaluated through plaque neutralization reduction tests (PNRT) (15). However, this is controversial as other researchers have not been able to demonstrate such a relationship (13). Moreover, a positive correlation has been observed between PB frequency and virus-specific IgG levels in symptomatic and asymptomatic COVID-19 individuals (16). Still, even a robust humoral response apparently fails to protect against severe COVID-19 (17). These discrepancies could be due to biological factors related to the lower potency of the antibodies, delay in the kinetics of appearance and state of inflammation, among others (18).

As concerns about immunity against SARS-CoV-2 persist, it becomes crucial to elucidate aspects of B cell induction, activation and differentiation under natural infection and its association with disease course. In this study, we aimed to characterize the dynamics of B cell subsets and neutralizing responses in COVID-19 participants according to disease severity during a one month of follow-up.

## Materials and methods

### Study population

We recruited a Colombian cohort of 71 individuals, over 18 years old from *Hospital Alma Mater de Antioquia*, *Hospital Universitario San Vicente Fundación, Hospital Digital Living Lab* and the *Grupo Inmunovirología* of the Universidad de Antioquia, Medellin, Colombia, with a positive RT-PCR for SARS-CoV-2. The individuals were enrolled between November 2020 and July 2021, and each of them was assigned to one of the four clinical status groups: i) asymptomatic (n=20), ii) symptomatic (n=20), iii) hospitalized in ward (n=11), and iv) ICU (n=20). Most asymptomatic individuals did not exhibit any signs or symptoms of COVID-19. However, we also considered asymptomatic those who reported nonspecific symptoms of concise duration (*i.e.* less than two days) but also symptoms associated with chronic conditions or related to climatic or air pollution conditions. We considered fever, chills, dyspnea, anosmia and diarrhea the most suggestive symptoms of COVID-19, according to what had been reported in the literature (19, 20). If our participant presented any of these, it was immediately classified as a symptomatic group. Symptomatic individuals were those with mild-moderate signs or symptoms related to COVID-19 who were not treated at the hospital. Hospitalized patients (hereon referred only as “hospitalized”) were defined as those who, due to the nature of their disease, required hospital admission for treatment but only required non-invasive oxygen support with high or low flow systems. Finally, ICU patients were those who suffered from severe disease and required treatment at the intensive care unit, as well as mechanical ventilation.

All participants had their first nasal swab and blood sample taken at the time of inclusion in the study. For asymptomatic participants, samples were taken a maximum of 5 days after a positive RT-PCR SARS-CoV-2 test or after close contact with COVID-19 positive individuals. For symptomatic patients, samples were withdrawn at around 5 days after the onset of symptoms, preferably under seven days. Both, hospitalized and ICU participants, were sampled within the first three days of hospitalization at their respective locations. For all participants, further blood samples were obtained at 7 ± 2 and 30 ± 5 after the inclusion, although some patients were lost during follow-up, in particular at the last moment of sampling, due to demise, hospital discharge or dissent. By day 30, in the asymptomatic group we were able to follow up eight participants; whereas in the hospitalized in ward and ICU study groups, six and three participants were follow-up, respectively. There were no losses in the symptomatic group.

The exclusion criteria were as follows: children, pregnant women, patients with acute respiratory infection who did not meet the COVID-19 case criteria according to the Colombian Ministry of Health, participants who did not accept participation or follow-up and those vaccinated against COVID-19. All participants provided written informed consent, and the study was approved by the Bioethics Committee of the Faculty of Medicine, Universidad de Antioquia (certificate of approval No.012)

### Serum and cell isolation

EDTA-whole blood samples were collected, and peripheral blood mononuclear cells (PBMCs) were isolated through a blood density gradient using the Histopaque reagent (Sigma-Aldrich) according to the manufacturer’s instructions. Isolated PBMCs were cryopreserved until the day of the test. Participants sera were collected using serum separator tubes. After centrifugation, samples were stored at −80°C until testing.

### Flow cytometry

The phenotype of B cells was evaluated by flow cytometry on 0.8x10^6^ cells stained with a viability dye (1:1000, Fixable Viability Dye, ThermoFisher Scientific, USA) along with antibodies specific against CD27 (clone: M-T271, BD Biosciences, New Jersey, USA), CD19 (clone: HIB19, BD Biosciences), CD20 (clone: 2H7, BD Biosciences), CD24 (clone: ML5, BD Biosciences), IgD (clone: IA6-2, BD Biosciences), CD38 (clone: HIT2, BD Biosciences), IgG (clone: G18-145, BD Biosciences), IgM (clone: G20-127, BD Biosciences) and CD40 (clone: 5C3, ThermoFisher Scientific) for 30 minutes at 4°C protected from light. Samples were acquired on a LSR Fortessa (BD) flow cytometer, and the results were analyzed using the FlowJo v.10.8 software. Dimension reduction of down-sampled and concatenated data sets was performed using the FlowJo plugin for the algorithm t-SNE (T-distributed Stochastic Neighbor Embedding).

### Immunofluorescence assay

A fluorescence immunoassay was performed to determine the presence of anti-SARS-CoV-2 antibodies in the serum of enrolled participants. For this purpose, 20 µL of serum at 1:20 dilution with PBS were added into slides containing Vero E6 cells infected with B.1 lineage of SARS-CoV-2 and then were incubated for 30 minutes at 37°C. Serum from a convalescence patient pre-analyzed was used as a positive control, and non-infected cells were used as mock. Later, the slides were washed twice with PBS, considering that the whole slide had to be submerged and was allowed to air dry. Then, 20 µL of goat anti-human IgG (Fc specific)- FITC antibody (Sigma-Aldrich) at 1:40 dilution with PBS was added and incubated for 30 minutes using a Humidifying Chamber. Finally, immunofluorescence was assessed by microscopy in an Axio Vert.A1™ (Zeiss, Oberkochen, Germany)

### Neutralizing antibody assay

The neutralizing antibodies titer from serum was determined using the plaque reduction neutralization test (PRNT) and reported as a neutralizing endpoint. Vero E6 cells (1.1 x 10^5^ cells per well) were seeded in 24-well tissue culture plates and incubated at 37°C and 5% CO2. The next day, 100 µL of heat-inactivated sera (quadruple dilutions from 1:20 to 1:20480) were mixed with 80 PFU/0.1 mL of SARS-CoV-2 (B.1 lineage) in microcentrifuge tubes and incubated for one hour at 37°C. We used virus in absence of serum as a viral control, and serum without SARS-CoV-2 as a mock control. Then, the mixtures were added by duplicate to Vero E6 monolayers and incubated at 37°C, 5% CO2 for 1h. Later, the inoculum was removed and replaced by 1 mL of semisolid medium (1.5% carboxymethyl cellulose, 2% fetal bovine serum, 1% streptomycin, and DMEM 1X) and incubated for 3-4 days. Finally, semisolid media was removed, monolayers were washed twice with PBS, fixed and stained with 4% formaldehyde/1% crystal violet for 30 min, and washed twice with PBS. The neutralizing titers were reported as the inverse endpoint dilution of serum that could neutralize 50% of viral plaque formation (PNRT_50_).

### Statistical analysis

We conducted a mixed-effects model with the Geisser-Greenhouse correction to compare the frequencies of different B cell subsets as well as the neutralizing titers among the severity groups over time. Then, a Tukey’s multiple comparisons test was applied. Data are presented in percentages, median and interquartile ranges (IQR) as they correspond. The immunofluorescence assay results were analyzed with a Fisher’s exact test. Correlation analyses were calculated using the Spearman rank-order correlation coefficient. We considered p-values < 0.05 as statistically significant. Statistical analysis was performed using GraphPad Prism version 9.0.1, San Diego, California, USA (GraphPad Software).

## Results

### Sociodemographic and clinical characteristics

The median age was higher in hospitalized (55, IQR 41 to 64) and ICU (64, IQR 55 to 73) groups compared to asymptomatic (30, IQR 25 to 43) and symptomatic (34, IQR 23 to 51) groups. The body mass index (BMI) also had similar behavior, being higher in hospitalized (27.7, IQR 24.0 to 34.2) and ICU (27.2, IQR 25.6 to 30.7) groups and their medians spotted within the overweight range. Most participants in different groups were women, except in the hospitalized group where men prevailed (**Table 1**). All participants included in our study were Hispanic/Latino adults.

**Table 1:** Baseline sociodemographic and clinical characteristics.

Characteristic	Asymptomatic	Symptomatic	Hospitalized	ICU
	n = 20	n = 20	n = 11	n = 20
Age – yr (IQR)	30 (25-43)	34 (23-51)	55 (41-64)	64 (55-73)
Male sex - no. (%)	6 (30)	5 (25)	8 (73)	9 (45)
Weight – Kg (IQR)	66.5 (62.3-71.5)	66.5 (53.5-79.0)	80.0 (68.0-85.0)	70.0 (65.3-79.5)
BMI (IQR)	23.9 (22.1-28.0)	23.7 (22.3-26.8)	27.7 (24.0-34.2)	27.2 (25.6-30.7)
Signs and symptoms - no. (%)				
Fever	0 (0)	5 (25)	8 (73)	13 (65)
Chills	0 (0)	13 (65)	3 (27)	1 (5)
Dyspnea	0 (0)	3 (15)	10 (90)	20 (100)
Fatigue	1 (5)	16 (80)	5 (45)	14 (70)
Dry cough	1 (5)	10 (50)	5 (45)	9 (45)
Myalgia	2 (10)	10 (50)	4 (36)	3 (15)
Headache	3 (15)	15 (75)	3 (27)	3 (15)
Anosmia/ageusia	0 (0)	10 (50)	3 (27)	4 (20)
Odynophagia	2 (10)	16 (80)	3 (27)	2 (10)
Rhinorrhea	2 (10)	8 (40)	3 (27)	3 (15)
Nausea/vomiting	0 (0)	5 (25)	5 (45)	2 (10)
Diarrhea	0 (0)	5 (25)	3 (27)	4 (20)
Coexisting conditions - no. (%)				
Hypertension	0 (0)	3 (15)	6 (55)	7 (35)
Diabetes	0 (0)	1 (5)	2 (18)	7 (35)
Asthma	2 (10)	1 (5)	0 (0)	1 (5)
COPD	0 (0)	0 (0)	0 (0)	2 (10)
Dyslipidemia	0 (0)	1 (5)	3 (27)	2 (10)
Hypothyroidism	2 (10)	1 (5)	0 (0)	3 (15)
Any condition*	6 (30)	6 (30)	7 (64)	16 (80)
Laboratory values (median and range)				
D-dimer level (µg/mL)	–	–	1 (0.3 – 1.8)	1.4 (0.61 – 1.8)
LDH (U/L)	–	–	421 (195.2 – 509.1)	709.2 (335 – 509.1)
Ferritin (μg/L)	–	–	1111.1 (44.5 – 1500.9)	1160.7 (193.9 – 1500.9)
CPR (mg/L)	–	–	9.3 (1.3 – 28.5)	15.5 (4.1 – 28.5)
Creatinine (mg/dL)	–	–	0.9 (0.7 – 1.3)	0.9 (0.5 – 1.3)
White blood cells — x10^3^ per mm3	–	–	9 (3.4 – 19.6)	11 (3.7 – 19.6)
Relative neutrophil count	–	–	79 (54.1 – 92.4)	87.5 (58.2 – 82.4)
Relative lymphocyte count	–	–	13.3 (3.2 – 35.7)	7.1 (2.2 – 35.7)
Platelets — x10^3^ per mm3	–	–	275 (56 – 528)	226.5 (77 – 528)

ICU: intensive care unit, IQR: interquartile range, BMI: body mass index, COPD: Chronic Obstructive Pulmonary Disease, LDH: lactate dehydrogenase, CPR: C-reactive protein.

*Any condition refers to the number of individuals in each group who had at least 1 coexisting condition, including those already listed in this table and those not listed because of their low relevance, which was a psychiatric disease, chronic kidney disease, atopy, and rheumatism.

Some individuals in the asymptomatic group had non-specific symptoms that did not last longer than three days, such as fatigue (5%), dry cough (5%), headache (15%), odynophagia (10%) and rhinorrhea (10%). These symptoms, if experienced for said short period, did not exclude participants from the asymptomatic group because they could be attributed to other conditions and are common even in healthy individuals without an undergoing infection. The most common signs and symptoms in the symptomatic group were fatigue (80%), odynophagia (80%), headache (75%) and chills (65%). Meanwhile, fever and dyspnea were the most frequent clinical manifestations among patients admitted to the hospital. Fever was present in 73% and 65% of individuals in hospitalized and ICU, respectively. It is worth noting that 90% of hospitalized individuals had dyspnea and required non-invasive oxygen support, and all individuals in the ICU group had severe dyspnea and required invasive mechanical ventilation (**Table 1**). The survival rate in the hospitalized group was 90.1%, but fell to a mere 35% in the ICU group.

Measurements from admission laboratories were collected from the medical records of hospitalized and ICU patients. The medians of D-dimer level, lactate dehydrogenase (LDH), ferritin and C-reactive protein (CRP) were higher among ICU patients compared to their hospitalized counterparts, and ICU patients displayed a more evident neutrophilia and lymphopenia (**Table 1**).

### The B cell response is heterogeneous among COVID-19 patients

**Figure 1:**
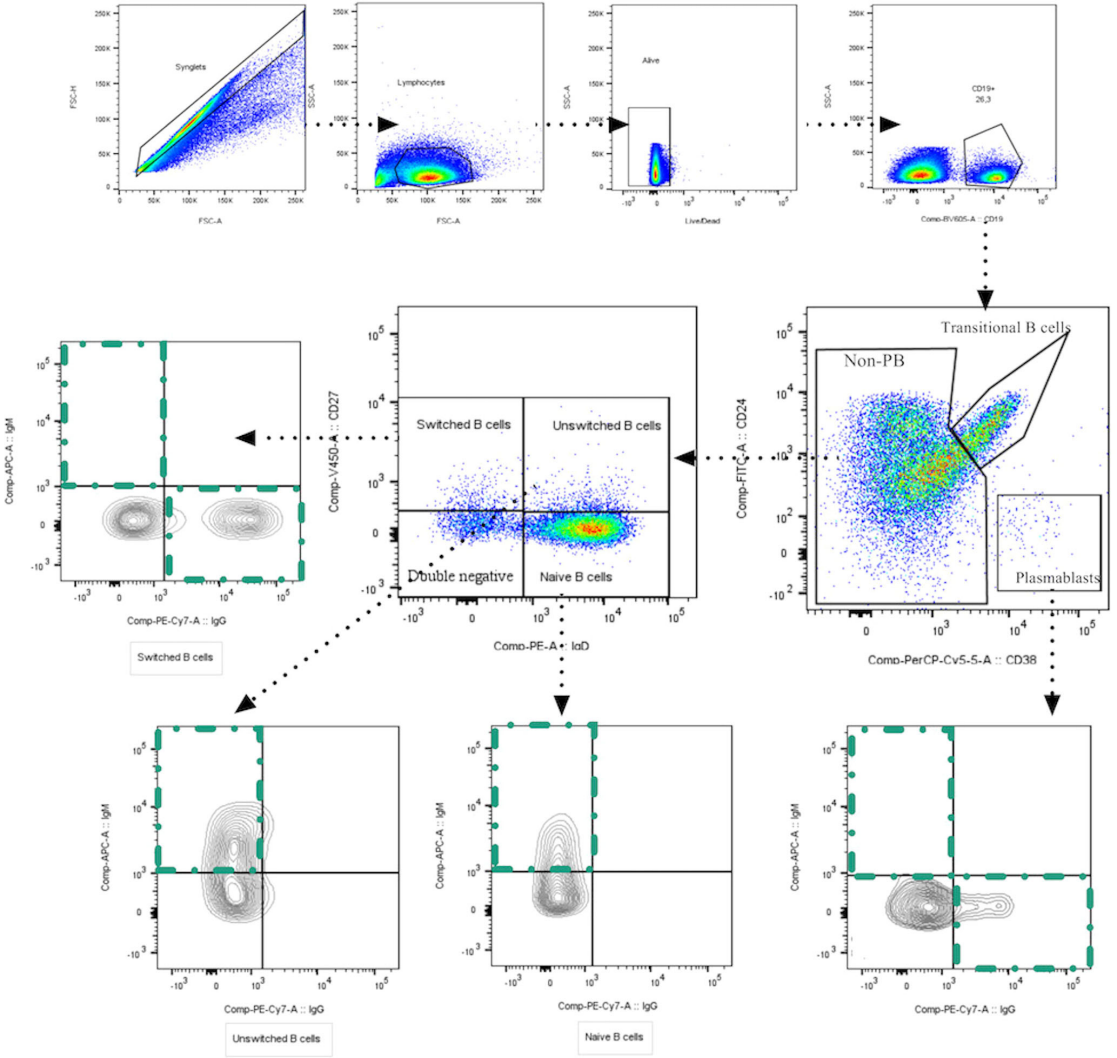
Gating strategies to identify B cell subpopulations. Representative plots show a singlets gate identified by FSC-H vs. FSC-A parameters followed by FSC vs. SSC plot to identify the total lymphocyte population. Live cells were selected based on negative live/dead staining. Subsequently, CD19+ B cells were set, and several B cells subpopulations were identified based on the expression of surface markers: transitional B cells (CD24^+^CD38^High^); plasmablasts (CD24^-^CD38^High^) that express IgM^+^, IgG^+^ or IgG^-^/IgM^-^; naïve B cells (CD24^+^IgD^+^CD27^-^); memory B cells (MBCs, CD24^+^CD27^+^IgD^+^) that could be classified in IgM-expressing unswitched and IgM-negative memory B cells (only IgD^+^); memory B cells CD27^+^IgD^-^ that express IgM (pre-switched memory cells), and that express IgG (switched memory cells); and double negative B cells (DN) (CD24^+^CD38^+^CD27^-^IgD^-^IgG^-^).

**Figure 2:**
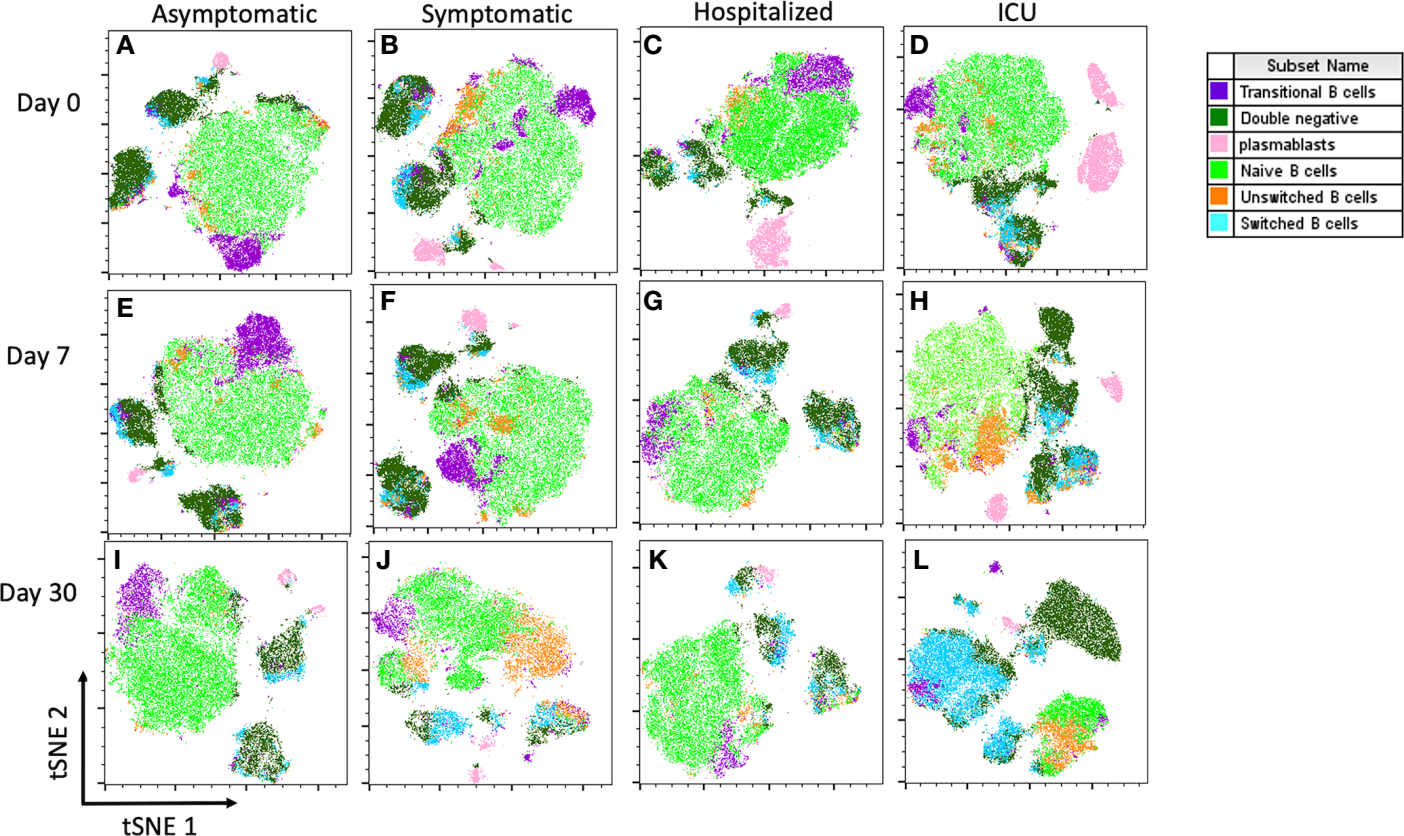
t-SNE analysis of B cell subsets in each clinical group at days 0, 7 and 30. Each density plot is derived from random sampling of about 3.000 single events from the concatenated individual cytometry data according to the severity of COVID-19 participants. **(A-D)** samples at day 0, **(E-H)** samples at day 7, and **(I-L)** samples at day 30. *L. was concatenated of only three participants.

We assessed the dynamic changes of several B cell subpopulations in each group and on three time-points through multiparametric flow cytometry. The gating strategy used to identify B cell subpopulations is shown in **Figure 1**. t-SNE plots show the composite samples for all assessed fluorescence parameters, and cell clusters are depicted in a Cartesian space for each severity group during the follow-up. In general, great heterogeneity was observed among B cell subsets in different groups in a time-dependent fashion. In asymptomatics, we detected a low percentage of PBs at the time of recruitment, but in the other groups there was enrichment of PBs at day 0, which then waned throughout the 30 days of follow-up (**Figure 2**). Naïve B cells seem to remain unchanged during follow-up, except at day 30 in ICU patients, where a decrease in this population was observed. Other interesting findings were the increase of unswitched MBCs in symptomatic patients and the increase in switched MBCs in ICU over time (**Figure 2**).

### Hospitalized but not ICU patients achieve and maintain a higher proportion of IgG^+^ plasmablasts over time

**Figure 3:**
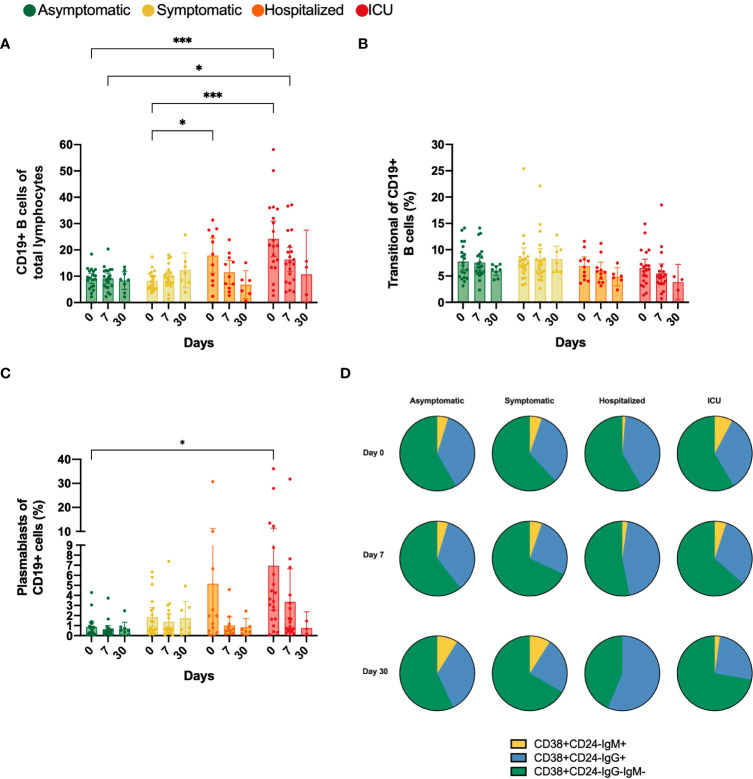
Percentage of CD19^+^, transitional and plasmablasts (PB) B cells and proportion of IgM^+^, IgG^-^ and IgG^-^IgM^-^ plasmablasts. Bar charts illustrate the percentage of **(A)** CD19^+^ cells, **(B)** Transitional and **(C)** PB cells, represented by mean and confidence interval (CI, 95%). **(D)** Parts of whole diagrams depict the proportion of either IgM^+^, IgG^+^ or IgM^-^IgG^-^ expression in the PB population. All graphs represent asymptomatic, symptomatic, hospitalized, and ICU groups on days 0, 7 and 30 after recruitment. Data were analyzed using a mixed-effects model with the Geisser-Greenhouse correction with Tukey’s multiple comparisons testing between groups. A p-value < 0.05 was considered significant. *p < 0.05, ***p < 0.001.

Initially, the CD19 biomarker for B cells was evaluated in each group. We observed a significant increase in the frequencies of CD19^+^ cells at day 0 of recruitment time in the hospitalized (17.77%) and ICU (24.21%) patients compared to asymptomatic (9.13%) and symptomatic (8.24%) groups (**Figure 3A**). However, in hospitalized COVID-19 patients, the B cell frequencies seemed to wane when comparing time-points during follow-up. We did not observe any significant change in the frequency of transitional B cells among study groups (**Figure 3B**).

Similarly, the frequency of PB was significantly higher at the first sampling time in the ICU compared to the asymptomatic group (mean 0.90% vs 6.95%, p=0.0437). Although it was not significant, the frequency of PB also tended to be higher in the hospitalized group (**Figure 3C**). However, the frequencies of PB tended to decline over time in both hospitalized and ICU groups. Additionally, we analyzed the IgM and IgG expression in the PB population and noticed that in all study groups and during the total follow-up time, the IgM^-^IgG^-^ PB were the predominant subset, which might correspond to IgA^+^ PBs. Moreover, in both groups requiring hospital care, the IgM^+^ PBs tend to decrease over time, while the IgG^+^ PB proportion increased, mainly in the hospitalized group (**Figure 3D**).

### Hospitalized and ICU individuals exhibit significant changes in the pool of naïve and memory B cells

**Figure 4:**
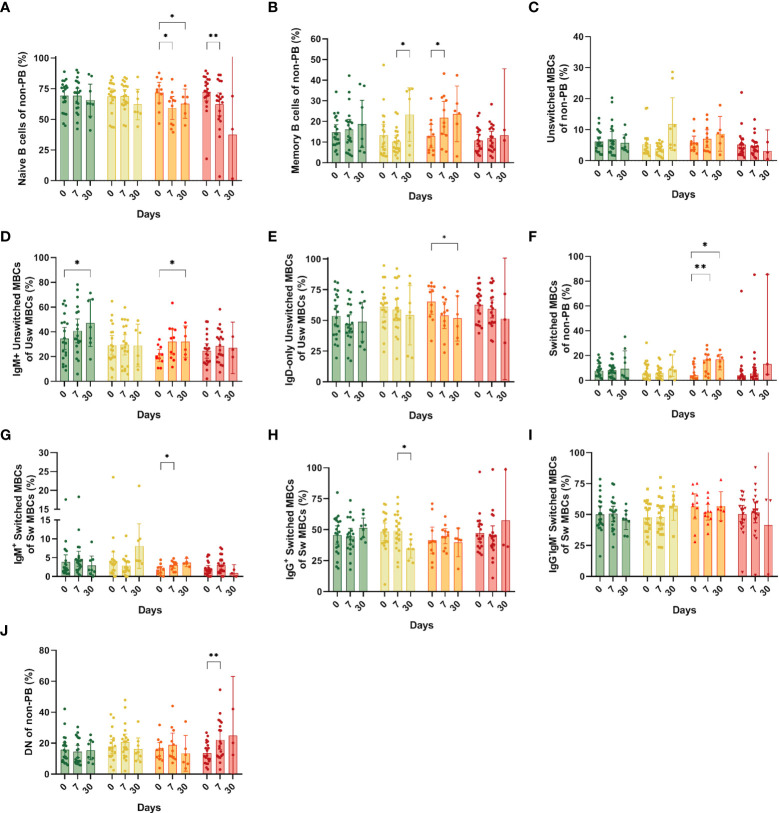
Frequencies of memory B cell subsets (MBCs) in the different study groups at days 0, 7 and 30 after recruitment. **(A)** Naïve B cells; **(B)** Total MBCs; **(C)** Unswitched MBCs; **(D)** IgM^+^ unswitched MBCs; **(E)** IgD-only (IgM^-^IgG^-^) unswitched MBCs; **(F)** Switched MBCs; **(G)** IgM^+^ switched MBCs (pre-switched); **(H)** IgG^+^ Switched MBCs; **(I)** IgG^-^IgM^-^ Switched MBCs; **(J)** Double negative from the non-PB region. Bars show mean and confidence interval (CI, 95%). Data were analyzed using a mixed-effects model with the Geisser-Greenhouse correction with Tukey’s multiple comparisons testing between groups. A p-value < 0.05 was considered significant. *p < 0.05, **p < 0.01.

We investigated whether other cells could explain the changes observed in the pool of CD19^+^ B cells, and found a significant reduction in the naïve B cell population in the hospitalized group at days 7 and 30 compared with day 0 (mean: 59.3% and 62.82% vs. 71.81%, respectively, p=0.03 and p=0.03) and in the ICU group at day 7 compared to day 0 (mean: 32.3% vs. 62.25%, p= 0.03) (**Figure 4A**). Next, we analyzed the behavior and dynamics of MBCs. A significantly increase in MBC frequencies (defined as CD27^+^/IgD^-^ or IgD^+^) at day 7 compared to day 0 was observed in the hospitalized group (mean: 12.8% vs. 28.72%, p=0.03) (**Figure 4B**). We also observed an augmented frequency of MBCs in the symptomatic group at day 30, which had a statistically significant difference with that of day 7 (**Figure 4B**). Although we did not see statical differences in the total unswitched MBCs (**Figure 4C**), when assessing IgM^+^ unswitched MBCs, we also observed an increase in their frequency at day 30 compared with day 0 in both asymptomatic (mean: 47.23% vs. 34.64%, p=0.04) and hospitalized patients (mean: 20.95% vs. 32.13%, p=0.05) (**Figure 4D**). In the latter, we saw a decrease in frequency of IgD-only unswitched cells at day 30 compared to day 0 (mean: 51.68% vs 65.29%, p=0.03) (**Figure 4E**).

Regarding class-switched MBCs, we only noticed changes in the hospitalized group with a surge at days 7 and 30 compared to the day of recruitment (mean: 14.63% and 14.99% vs. 7.05%, p=0.01 and p=0.04) (**Figure 4F**). Among switched MBCs, we also found an increase of IgM^+^ (pre-switched memory cells) in the hospitalized group at day 7 compared to day 0 (mean: 2.99% vs. 1.81%, p=0.02) (**Figure 4G**). A significant reduction in the frequency of IgG^+^ switched B cells was observed in the symptomatic group at day 30 compared to day 7 (mean: 49.11%vs.34.80%, p=0.01) (**Figure 4H**). Finally, B cells that are double negative for CD27 and IgD were expanded only in ICU individuals at day 7 compared to day 0 (22.17% vs. 13.65%, p=0.003) (**Figure 4J**). No other significant differences were observed among the study groups.

### COVID-19 patients in the hospital setting may elicit a strong neutralizing antibody response

**Figure 5:**
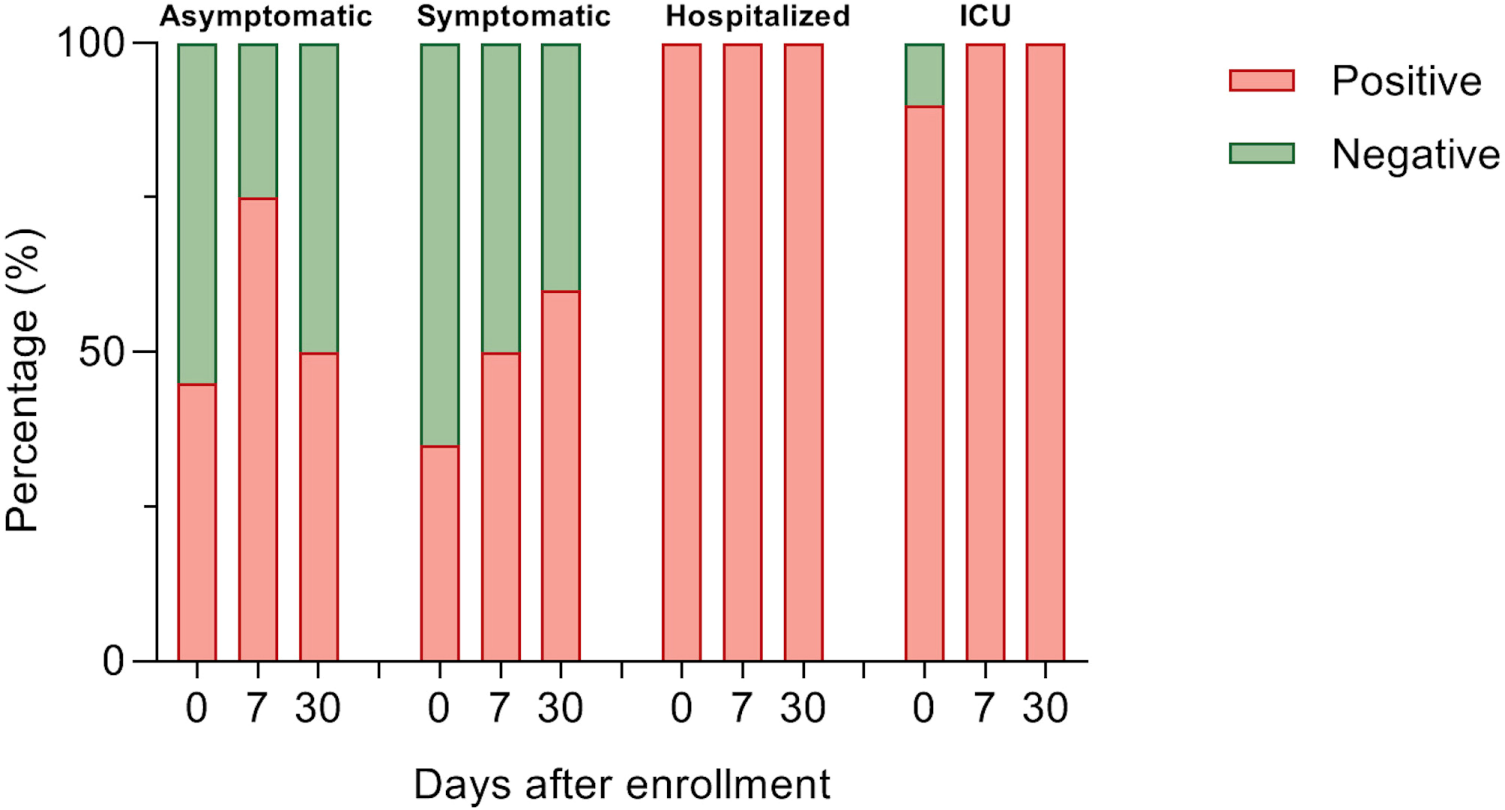
Detection of anti-SARS-CoV-2 total IgG antibodies. Percent of participants with anti-SARS-CoV-2 IgG in 1:20 serum using immunofluorescence assay in each study group over a month of follow-up. Data were analyzed with Fisher’s exact test.

**Figure 6:**
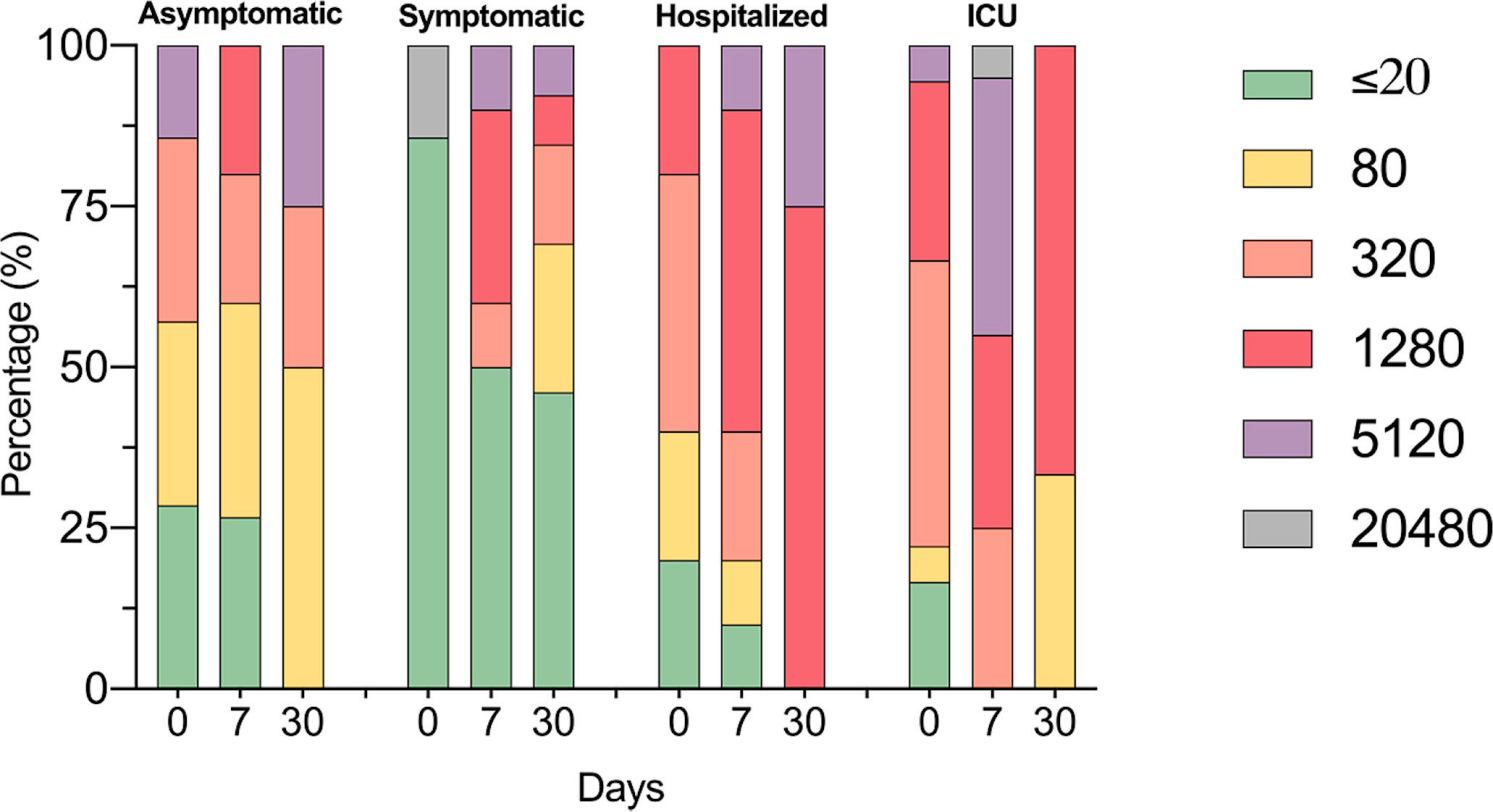
Identification of SARS-CoV-2 neutralizing antibodies. The bar graph shows the percentages of COVID-19 participants according to neutralizing antibodies titers by PNRT_50;_ follow-up for one month.

For determining antibody titers, first we performed an immunofluorescence assay (IFA) which detects total IgG anti-SARS-CoV-2 in serum at 1:20 dilution. As shown in **Figure 5**, the proportion of individuals with a positive IFA was higher in both groups admitted to the hospital compared to asymptomatic and symptomatic individuals at all-time points assessed. Then, only participants with a positive IFA were evaluated for neutralizing antibodies (NAbs) by a plaque reduction neutralization test (PNRT_50_). Although both asymptomatic and symptomatic groups showed a NAb response, the proportion of individuals with PNRT_50_ equal to or higher than 1:1280 tended to be higher in hospitalized and ICU patients and, strikingly, 86% of symptomatic individuals had titers equal to or below 1:20 at day 0 (**Figure 6**). It is worth noting that even in asymptomatic individuals, titers as high as 1:5120 were reported, and at the same time, some ICU patients also had low NAb responses, although the trend of higher NAb titers as severity increased was the rule. After 7 days, the ICU group had NAb titers markedly higher than the asymptomatic group (3536 vs. 348, p=0.026). Through time as well, mean NAb titers were higher as the severity of COVID-19 increased, howbeit some differences between groups showed a trend but not were not statistically significant, and the NAb response was highly variable. At 30 days, the NAb titers of the individuals who did not attend a hospital were lower than those who did require hospital care. The hospitalized and ICU participants maintained higher NAb titers at day 30, although there were no significant differences among the groups at this time point (**Figure 6**).

**Figure 7:**
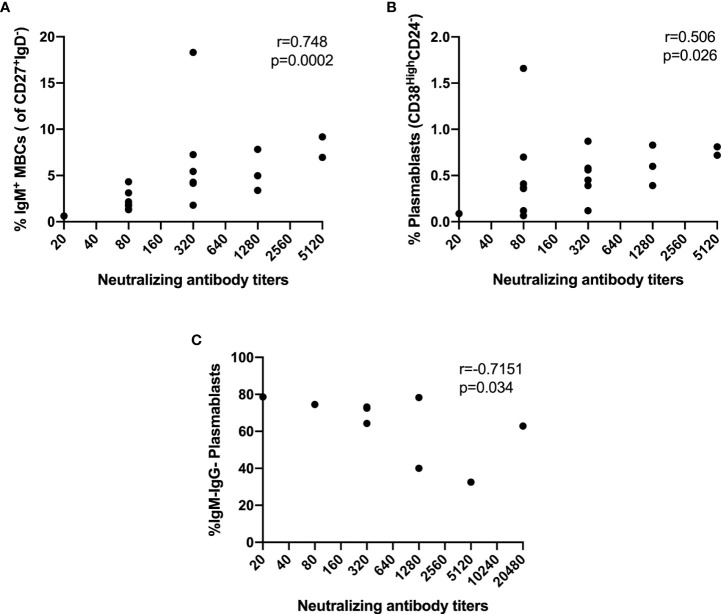
Correlations between NAbs and frequencies of B cells subsets. **(A)** IgM^+^ Switched MBCs from asymptomatic, **(B)** PB from asymptomatic, **(C)** IgM^-^IgG^-^ PB from symptomatic. Spearman’s correlation coefficient (r) and p-value are shown.

Then, we decided to explore the relationship between B cell subsets and NAb response. Interestingly, the NAb titers in asymptomatic individuals correlated strongly with the percentage of IgM^+^ switched MBCs (r=0.748, p=0.0002) (**Figure 7A**) and moderately with PBs (r=0.506, p=0.026) (**Figure 7B**). Furthermore, in symptomatic individuals, we found a strong negative correlation between the NAb titers and IgM^-^IgG^-^ PBs (r=-0.7151, p=0.034) (**Figure 7C**). We did not find correlations between NAb titers and B-cell subsets in hospitalized and ICU groups.

### Severity in COVID-19 disease is related to inflammation state

**Figure 8:**
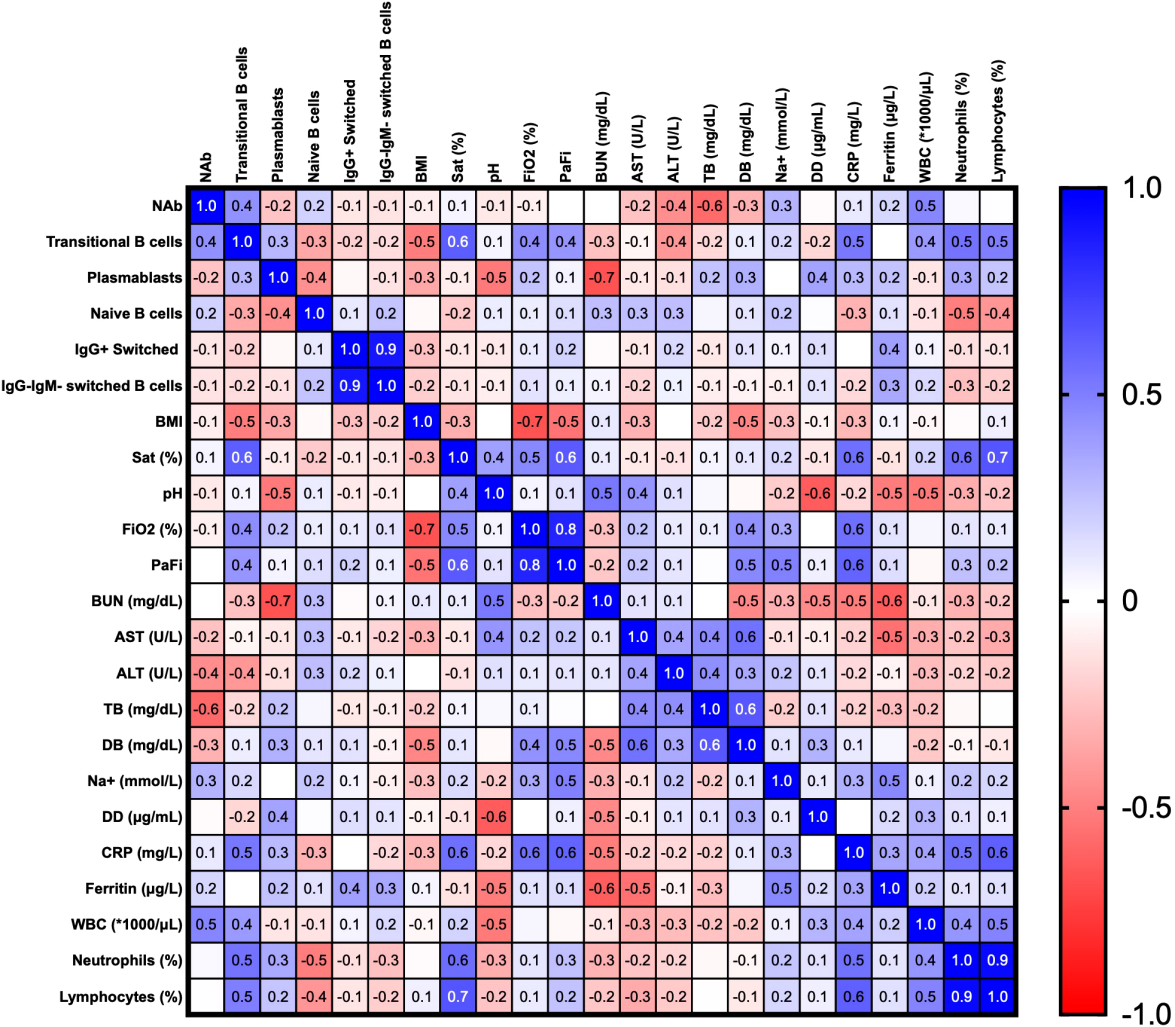
Correlation matrix of clinical parameters and B cells subsets in the hospitalized and ICU patients at the recruitment time. Spearman’s correlation coefficients are plotted, and the scale colored reveals the strength of correlations (shades of red= negative, shades of blue= positive). *NAb: neutralizing antibodies, BMI: body mass index, Sat (%): oxygen saturation, FiO_2_: fraction of inspired oxygen, PaFi: ratio of arterial oxygen partial pressure to fractional inspired oxygen, BUN: blood urea nitrogen, AST: aspartate aminotransferase, ALT: alanine transaminase, TB: total bilirubin, DB: direct bilirubin, DD: D-dimer test, CRP: C-reactive protein, WBC: white blood cells.

Finally, we established correlations between clinical parameters, different B cell subsets and neutralizing responses in the hospitalized and ICU groups at the recruitment time, using the correlation matrix shown in **Figure 8**. A positive correlation was found among transaminases and transitional B cells, PBs, naïve and IgM^-^IgG^-^ switched MBCs subsets. Moreover, acute phase reactants such as CRP, D-dimer (DD), and ferritin were positively correlated with WBC counts and relative counts of neutrophils, but negatively with those of lymphocytes. WBCs and neutrophil counts were themselves positively correlated (r=0.54, p=0.002), with a negative correlation with lymphocyte counts (r=-0.67, p= 0.00009), highlighting the common presentation of neutrophilia and lymphopenia in severely ill patients. Interestingly, a slight negative correlation was observed between BMI and NAb titers. (r=-0.44, p=0.02) (**Figure 8**).

## Discussion

We characterized the dynamics of B cell subsets and neutralizing response in 71 participants with different clinical stages of COVID-19. As expected, the frequencies of some of the B cell subsets were modulated according to disease severity and changed over time. Generally, we observed an increase in the frequency of CD19^+^ B cells on the first day of recruitment in most severe cases of COVID-19 compared with mild cases, which is similar to what has been previously reported (8, 17), suggesting that at the initial moment of recruitment, a clonal expansion antigen-driven was observed (21). Going into specifics, transitional B cells are a stage of immune development between immature B cells at the bone marrow and circulating mature B cells. Some authors have reported a decay in the transitional subset as COVID-19 worsens, and it has also been noted that this subset is waned in COVID-19 patients compared to healthy individuals (8, 11). In contrast to these reports, we did not find significant differences in transitional B cells among study groups, despite the fact that this subset showed a tendency to decrease in some of our ICU patients.

While some studies have found that there are no significant differences in naïve B cell frequency between mild and severe COVID-19 patients, we found that relative values of naïve B cells seem to be affected in severe cases and decrease over time, while this B cell population seems unaffected in mild patients throughout the follow-up (8, 22). In fact, other studies have shown similar results to our findings, as some authors have described an altered frequency of B cell subsets in COVID-19 patients over time, and a particular increase in naïve B cells in severe patients compared to milder cases, which could be consistent with the development of humoral responses, as others suggested (23). Moreover, a decrease at day 7 and 30 of Naïve B cells in severe COVID-19 suggest a differentiation towards PB or MBCs (10, 24).

Early after antigen encounter, Naive B cells experience a process of expansion and differentiation towards PB, germinal center B cells and MBCs (21). PB produces antibodies with a low level of somatic hypermutation outside the follicle, and in severe COVID-19 cases has been observed a robust extrafollicular response. In our study, the PB subset peaked in acute stages of SARS-CoV-2 infection, with a conspicuous expansion in critical COVID-19 patients, and then decayed over time. This same behavior has been observed throughout several reports, both in response to natural infection and vaccines (25–29). In addition, among the participants of our study, all groups had a higher proportion of IgG^-^IgM^-^ PBs, which, as hypothesized by several authors, probably corresponds to IgA^+^ plasmablasts and might be crucial in mucosal viral clearance and neutralizing ability against SARS-CoV-2 (25, 30). However, we saw an increase in the frequency of IgG^+^ PBs in hospitalized patients over time, which was distinct from those of the other study groups (31). These results suggest a variable behavior of PBs that, in the context of Noval M et al.’s findings, could suggest a more efficient neutralization due to a broad isotypical spectrum of antibodies against SARS-CoV-2 (32). The peak and decay in PBs that we showcased might be related to infection persistence, as Gaebler et al. demonstrated a correlation between SARS-CoV-2 persistence in the intestinal tissue and the peak of PBs, hence playing an essential role in the evolution of antibody production (18). Although we do not analyze the response of SARS-CoV-2-specific B cells, several authors have reported an expansion of MBCs one-month post-symptom onset, and an increase of S-specific IgG^+^ switched MBC at five- months of follow-up. Moreover, the quality of response looks to be differential since individuals with non-severe COVID-19 have a phenotype associated with durable memory that is least frequently observed in severe COVID-19 (33). In agreement with those findings, severe COVID 19 induces an exhaustion phenotype on S1-specific IgG^+^ MBCs compared with healthy and recovered individuals (34). Although SARS-CoV-2-specific antibodies tend to wane over time, the persistence of MBC could supply its lost (35, 36).

MBCs can respond quickly to repeated challenges by differentiating between antibody-producing plasma cells or germinal center B cells (21). However, to date there is no consensus on the dynamics of MBCs in COVID-19 patients. Some authors have reported a decrease over time, both in class-switched and non-class-switched, with a particularly pronounced decrease in individuals with severe disease presentation (11, 22, 32, 37). In contrast, we observed an increase in total MBCs in mild and severe cases over time (**Figure 2**), although we were not able to establish the kinetics because the follow-up was short and the loss of individuals with severe clinical stages at day 30 was not negligible. In addition, IgM-unswitched MBCs were increasing in asymptomatic patients, which could be associated with healthy responses since no symptoms were developed (38). A similar picture was observed in hospitalized patients, with increased total switched, and IgM-switched MBCs, which was most likely related to viral antigen persistence (18). However, the functionality of these cells could be compromised since an increased expression of exhaustion markers has been reported in this population (34). Another scenario will be the vaccination of previously infected individuals who could benefit from only one shot of the vaccine, achieving a robust response from the MBC population since it can induce the expansion of new and persistent clones of MBC (39–41).

Some studies have reported that an inflammatory environment could drive the modulation of B cell subsets in critical COVID-19 patients (8, 42). In line with these studies, we found that hospitalized COVID-19 patients displayed a positive correlation between some clinical parameters and changes in B cell subsets. Also, in line with the findings from Cervantes-Díaz et al., ICU patients had an increase of double negative B cells (CD24^+^CD38^+^CD27^-^IgD^-^IgG^-^), which was previously reported to be modulated by both, pro- and anti-inflammatory signatures (43).

In our study, we found a moderate correlation between the production of NAbs and the expansion of PB in asymptomatic individuals, which could be associated with a protective role in the clinical course of infection. However, this correlation was not observed in hospitalized COVID-19 patients, despite high titers of neutralizing assays and a high frequency of PBs at day 0 of recruitment. This finding in hospitalized group could be associated with either a later sampling time during the infection compared to outpatients, or with an immune response established later on, although viral persistence and neutralizing antibody production by plasma cells in the bone marrow may also have a role (44). Furthermore, much has been reported on the heterogeneity of the NAbs response. Some authors have found that as the severity of the disease increases, so do the neutralizing titers in COVID-19 patients. One of the reasons that might explain the underlying variability is that the specific neutralizing response may be predominantly mucosal in some individuals, thereby having low NAbs titers in sera but high in mucosal fluids. Cervia et al. show that patients with mild symptoms have shown mucosal IgA titers with neutralizing capacity, even in the absence of SARS-CoV-2-specific sera antibody titers (45). Yet, other authors such as Woodruff et al. have shown that severe disease correlates with high neutralizing antibody production associated with extra-follicular activation and a repertoire of autoantibodies shared by autoimmune diseases, suggesting that the immune hyperactivation could elicit such a pathogenic humoral response (17).

Once SARS-CoV-2 infection has been established with severe manifestations, the neutralizing antibody response seems not to be as effective in controlling the virus. However, in survivors, NAb titers may help to protect against future infections due to the excellent relationship between neutralizing response and long-term protective immune memory (46). Although we did not establish the total duration of the neutralizing response, it might persist at least several months, as has been previously described (47).

Even though a strong NAb response is triggered in severe COVID-19, this response may not be helpful in controlling and clearing up the viral infection. Tang J. and collaborators observed that the NAbs from patients with fatal outcomes has lower affinity maturation when compared to survivors during the first month of hospitalization (48). A blockage in the antibody affinity maturation due to the loss of Bcl-6 expression in T follicular cells at germinal centers, and changes in the lymph node environment have been reported in autopsies of thoracic lymph nodes and spleens from deceased patients who succumbed to COVID-19 (48, 49).

There is still much left to understand regarding immune responses against SARS-CoV-2. One of the most worrying aspects is the emergence of variants and the potential impact that they will pose on humoral immunity, particularly since neutralizing antibody response is one of the most practical correlates of protection in the context of COVID-19 (50). Although we did not determine the virus linage for each sample and the recruitment of participants was done throughout various months, data from GISAID allow us to infer that variants circulating at recruitment time correspond to ancestral lineage B.1, Gamma, Lambda and Mu variants (51). Several reports have emerged, shedding light on a possible compromise of NAb response, and humoral responses in general, with the emergence of variants (52). Even though we did not carry out neutralizing assay using other variants, several authors have observed that the antibody response breadth in individuals with different clinical spectra of COVID-19 is reduced against variants of concern, even in severe COVID-19 (5, 53). Interestingly, the neutralizing activity against all variants increases after vaccination highlighting their importance as public health policies (54). It seems that highly genetically diverse variants stray away further from the boundaries of protection that both natural and vaccine-elicited immunity offer (5, 55). This seems particularly worrying in the context of our results since immunity already seemed to wane against the ancestral lineage. Still, more research must be conducted on the impact of other immunity mechanisms in this matter, particularly that of T cell responses.

Our study had some limitations. First, the recruitment time was taken as day 0 when the diagnosis was performed, so our participants had a few days of symptoms related to COVID-19 or a history of close contact with people confirmed for COVID-19. Although, all of them had a positive COVID-19 RT-PCR test within the last five days, we cannot assure the time length of the infection. Therefore, the onset of the effector immune mechanisms is variable among individuals. In addition, as indicated, a comparison with a baseline and a follow-up in a group of healthy donors were not carried out. On the other hand, the ICU group had a limited number of participants followed up until day 30 because most of them perished after day 7. In addition, we did not evaluate SARS-CoV-2-specific B cells which has shown a more accurate reflection of the humoral response (41), and more studies would be required to further corroborate the relationship between changes in SARS-CoV-2 specific and non-specific B cells.

Finally, our results suggest that COVID-19 could alter the frequencies of different B cell subsets. However, it seems that there are other immune mechanisms involved in the severity of the disease, so the determination of T cell dynamics, both functional and phenotypical, and the innate immune response, could be essential to have a better understanding of why some individuals evolve to a worse clinical course while others remain with asymptomatic or mildly symptomatic manifestations. In addition, we could establish that critical patients have a strong NAb response, suggesting that hospitalized COVID-19 patients who survive may have a robust memory immune response that could protect them from future reinfections. We only followed-up study participants for one month, thereupon, studies with more extended follow-up periods are required to clarify the duration of the memory immune response after natural infection, which is still subject to intense research and debate within the scientific community.

## Data availability statement

The original contributions presented in the study are included in the article/supplementary material. Further inquiries can be directed to the corresponding author.

## Ethics statement

The studies involving human participants were reviewed and approved by Bioethics Committee of the Faculty of Medicine, Universidad de Antioquia (certificate of approval No.012). The patients/participants provided their written informed consent to participate in this study.

## Author contributions

YM-C and AR-P contributed to data analysis, and manuscript preparation. AR-P and NA-M performed most of the experiments. MC-M, YM-C, JR-M and DM directed patient recruitment. MC-M and TJL contributed to writing and editing the manuscript. MTR oversaw conceptualization, project administration, critically review and editing of the manuscript. PAV was in charge of conceptualization, project administration, supervising data analysis and writing, as well as critically review and editing of the manuscript. All authors contributed to the article and approved the submitted version.

## Funding

This study was financed by Universidad de Antioquia and ICGEB Research Grant (CRP/20/015).

## Acknowledgments

The authors acknowledge the valuable contributions of Hospital Alma Máter de Antioquia, Hospital San Vicente Fundación, LivingLab and the volunteers. We want to thank Claudia Rugeles, Marlyn Dayna Sepúlveda, Maria Isabel Zapata and Carolina Montoya for their technical support during the development of the project. JR-M was supported by the Ministry of Sciences (MINCIENCIAS-Ministerio de Ciencia, Tecnología e Innovacion) from Colombia.

## Conflict of interest

The authors declare that the research was conducted in the absence of any commercial or financial relationships that could be construed as a potential conflict of interest.

## Publisher’s note

All claims expressed in this article are solely those of the authors and do not necessarily represent those of their affiliated organizations, or those of the publisher, the editors and the reviewers. Any product that may be evaluated in this article, or claim that may be made by its manufacturer, is not guaranteed or endorsed by the publisher.
